# Does the passage of seeds through frugivore gut affect their storage: A case study on the endangered plant *Euryodendron excelsum*

**DOI:** 10.1038/srep11615

**Published:** 2015-06-25

**Authors:** Shen Shikang, Wu Fuqin, Wang Yuehua

**Affiliations:** 1 School of life sciences, Yunnan University, Kunming No. 2 Green lake North road Kunming, Yunnan, 650091, the People’s Republic of China

## Abstract

Plant-frugivore mutualism serves an important function in multiple ecological processes. Although previous studies have highlighted the effect of frugivore gut passage on fresh seed germinability, no study has investigated the effect on seed storage after frugivore gut passage. We used the endangered plant, *Euryodendron excelsum*, to determine the combined effects of frugivore gut passage and storage conditions on the germination percentage and rate of seeds. In particular, three treatments that included storage periods, storage methods, and seed types were designed in the experiment. We hypothesized that seeds that passed through the gut will exhibit enhanced germination capacity and rate during storage. Results showed that the final germination percentage decreased in seeds that passed through the gut, whereas the germination rate increased during seed storage. Germination decreased in most types of seeds under both dry and wet storage after 6 months compared with storage after 1 and 3 months. The results suggest that after frugivore gut passage, *E. excelsum* seeds cannot form persistent soil seed bank in the field, and were not suitable for species germplasm storage. These finding underscore that seeds that passed through frugivore gut have long-term impact on their viability and germination performance.

Plant-frugivore mutualism serves a central function in a wide range of ecological processes, including species distribution^1^,^2^, population structure and dynamics^3^, species coexistence^4^,^5^,^6^, and biodiversity maintenance^7^,^8^,^9^,^10^. Plant-frugivore interaction is mutually beneficial because the dispersers receive resources (i.e., fruit pulp) while the plant seeds are dispersed away from the parent plant. This condition enables the avoidance of resource competition, risk of seed predation, and pathogen infection, while increasing seedling survival probability and occupancy of new habitat patches with favorable environmental conditions^8^,^11^,^12^. Successful dispersal can contribute to properous regeneration and restoration, as well as population spread and maintenance of genetic connectivity^12^,^13^, especially for endangered plants with limited distribution or small populations^14^,^15^. The effect of gastrointestinal tract ingestion by frugivores on the changes in seed traits, germination characteristics, and seedling establishment are important factors that influence successful recruitment and species diversity^10^,^11^,^16^. Although studies have highlighted the importance of frugivore gut passage in seed dispersal, as well as in seed germination characteristics among different groups both in natural ecosystems and artificial environments^11^,^17^,^18^,^19^, information regarding the long-term impact of plant-frugivore interaction on plant performance remains limited.

Frugivore gut passage of seeds provide immediate consequences for plants, such as seed germination, dormancy break, and escape from density and distance dependent mortality of seeds and seedlings^11^,^20^, as well as on long term plant performance, such as persistent seed bank, seed storage, and seedling emergence, which have been increasingly recognized^18^,^21^. Seed storage has been regarded as the most efficient method to store and maintain large pools of genetic diversity in plants and the preferred method of *ex situ* conservation^22^. Reinforcement of wild populations and reintroduction using artificial propagation from seed germination and seedling establishment may be valuable tools for conserving endemic and threatened species^22^,^23^. Biotic and abiotic factors during seed maturation and dispersal could affect seed viability and vigor in the subsequent storage processes^24^,^25^. To our knowledge, previous studies on plant-frugivore mutualism have mainly focused on the aspects of dispersal, fruit removal, post-feeding behavior, and effects on germination and seedling establishment^26^,^27^; no study has investigated the effect of seed passage through frugivore gut on seed storage.

*E. excelsum* is an endangered plant of the Theaceae family that is endemic to southern China. The major threats come from its small population size and high frequency of anthropogenic destruction^28^. The seed dispersion of *E. excelsum* is barochory and ornithochory in nature^28^,^29^. The species produce fleshy fruits that ripen between December to January when it is dry season in their natural habitat. Seeds dispersed by frugivores are needed to form a seed bank, which is utilized for germination and seedling emergence upon reaching the rainy season (from June to August), according to our field investigation from 2005 to 2009. However, seeds of *E. excelsum* that are dispersed by frugivores cannot always form an effective soil seed bank because of the high frequency of local anthropogenic disturbance^28^,^29^,^30^. Seed storage behavior and viability maintenance reflect the ability of the species to form soil seed bank that contributes to population regeneration and dynamics^31^,^32^. Therefore, we conducted a laboratory experiment to study the seed storage and seed viability maintenance after frugivore gut passage to simulate the principal process in the field. The results would help us to better understand the ability of the species to form a seed bank. Furthermore, seed storage behavior is also important to the species germplasm conservation^22^. Seeds collected in the natural habitat of the species come from fruits and bird droppings. Thus, we studied the effect of seed passage through frugivore gut on germinability during storage to understand the long term effect of plant-frugivore mutualism on seed viability maintenance. Given the influence of gut passage on seed germination is an enhancement effect rather than inhibition^11^,^27^, we hypothesized that seeds that pass through the gut have enhanced germination capacity and rate during storage.

To test our hypotheses, we designed a factorial experiment with three treatments that included storage periods (1, 3 and 6 months), storage methods (dry and wet), and seed types [seeds separated from fruits (SFF), seeds separated from droppings (SFD), seeds in intact fruits (SIF), and seeds in bird droppings(SID)]. We investigated the germination traits to evaluate the combined effect of seed passage through the gut and the storage conditions on the capacity and rate of germination.

## Results

### Germination percentage

**Figure 1:**
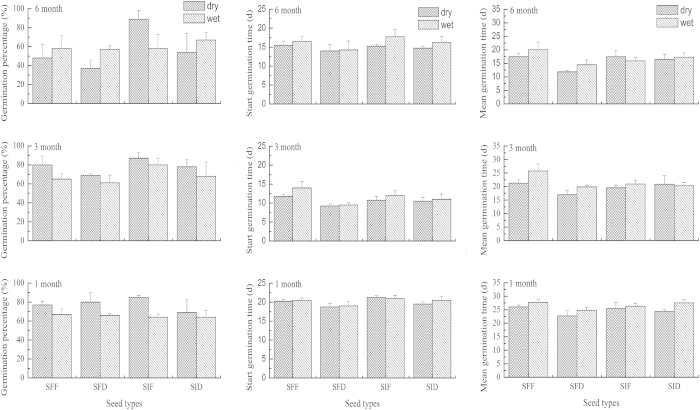
Germination percentage, start germination time (SGT) and mean germination time (MGT) of four seed types of *E. excelsum* under different storage conditions. Notes: SFF: seeds separated from fruits; SFD: seeds separated from droppings; SIF: seeds in intact fruits; SID: seeds in bird droppings.

The germination percentage of *E. excelsum* seeds varied across different storage conditions. The mean germination percentage ranged from 37% to 89% under different storage conditions after 1, 3, and 6 months. Most types of seeds had higher germination percentage after 3-month storage compared with 1-month storage, under both dry and wet storage conditions (Fig. 1). SIF under dry storage had the highest final germination percentage at each storage period. Germination of all types of seeds under both dry and wet storage after 6 months decreased compared with storage of 1 or 3 months, except for SIF under dry storage. Germination of SFD gradually declined with the increase in storage duration under both dry (from 80% to 37%) and wet (from 66% to 57%) conditions.

No significant difference in the germination percentage among the four types of seeds after 1-month storage (*F*_3,12_ = 2.313, *P* = 0.128) was observed under dry conditions. However, a significant difference in germination percentage of the four types of seeds after 3-month storage (*F*_3,12_ = 4.783, *P* = 0.02) was observed. The germination percentage of SFD was significantly lower compared with those of SFF and SIF (*F*_2,9_ = 7.883, *P* = 0.011). Germination percentage was also significantly different across the four types of seeds after 6-month storage, with significantly higher germination of SIF than that of the other three types of seeds (*F*_3,12_ = 10.625, *P* = 0.001). When seeds were stored under wet conditions, germination of SFF and SFD was significantly lower than that of SIF after 3-month storage (*F*_2,9_ = 8.209, *P* = 0.009). However, no significant difference in the germination percentage among the four types of seeds both after 1-month storage (*F*_3,12_ = 0.342, *P* = 0.796) and 6-month storage (*F*_3,12_ = 0.721, *P* = 0.558) was observed.

### Germination rate

Both SGT and MGT of the four types of *E. excelsum* seeds significantly decreased after 3-month storage (SGT: *F*_1,62_ = 611.332, *P* < 0.0001; MGT: *F*_1,62_ = 18.967, *P* < 0.0001) and 6-month storage (SGT: *F*_1,62_ = 153.317, *P* < 0.0001; MGT: *F*_1,62_ = 20.125, *P* < 0.0001), compared with those after 1-month storage. This result indicates the beneficial effect of storage on germination speed. The SGT and MGT of SFD were the lowest compared with those of the other types of stored seeds for each storage period (Fig. 1). Under both dry and wet conditions, the SGT of SFD was significantly lower than that of the other types of seeds, both after 1-month storage (dry: *F*_3,12_ = 10.429, *P* = 0.001; wet: *F*_3,12_ = 3.600, *P* = 0.046) and 3-month storage(dry: *F*_3,12_ = 7.000, *P* = 0.006; wet: *F*_3,12_ = 8.143, *P* = 0.003). However, no significant difference after 6-month storage (dry: *F*_3,12_ = 1.680, *P* = 0.224; wet: *F*_3,12_ = 2.567, *P* = 0.103) was observed. The MGT of SFD was also significantly lower compared with the other types of seeds at each storage period under dry storage (1 month: *F*_3,12_ = 3.467, *P* = 0.051; 3 month: *F*_3,12_ = 10.612, *P* = 0.001; 6 month: *F*_3,12_ = 4.102, *P* = 0.032) and wet storage (1 month: *F*_3,12_ = 5.521, *P* = 0.013; 3 month: *F*_3,12_ = 6.370, *P* = 0.008; 6 month: *F*_3,12_ = 7.410, *P* = 0.005).

### Factors influencing germination traits

The storage periods, methods, seed types, and their interactions significantly influenced the final germination percentage of *E. excelsum*. However, for the germination rate, only storage period had a significant effect on the SGT and only storage period and seed types significantly affected the MGT of seeds. The other factors and their interactions had no statistically significant effect on the SGT and MGT of *E. excelsum* (Table 1).

The best-performing model based on AICc values for germination percentage was the interaction of storage periods, methods, and seed types; none of the other tested models provided better results. Variation in the MGT of *E. excelsum* seeds was best explained by two models. The best model included storage periods, methods, and seed types. Five models substantially supported SGT. The best model only included storage periods as an explanatory variable (Table 2).

## Discussion

As highlighted by Figuerola *et al.*^21^ the effects of ingestion by vertebrates may exceed the period of fresh seed germination and may have long-term impact on plant performance, including seed storage behavior, seedling establishment, and plant growth. Numerous studies have highlighted the key functions of fruit-frugivore interaction in species coexistence and biodiversity maintenance^8^,^9^,^10^. In addition, seed storage has been recognized as the most effective method for germplasm preservation^33^,^34^. However, to the best of our knowledge, this study is the first to investigate the effect of gut passage on seed storage. Previously, we showed that germination of *E. excelsum* fresh seeds were unaffected by passage through the digestive tract of frugivores^29^. Thus, the present study has focused on the effects on seed storage, with emphasis on the effects of seed passage through the gut. Four types of seeds were germinated under different storage conditions during our study period.

Seed germination capability is often modified after seeds pass through the gut of frugivores^27^. The removal of pulp surrounding the seeds during gut passage has been regarded as an important mechanism that influences germination^18^,^35^,^36^. A series of studies has investigated the effect of seed consumption by frugivores and concluded that pulp removal can remove the inhibitory substance in the pericarp^36^. However, the effect of pulp removal on germination varies across different plant species and frugivores^11^,^17^. Germination is enhanced by pulp removal in some seeds^36^; however, others show lower germination percentage after pulp removal during gut passage compared with those that did not pass through the gut^17^. Meanwhile, in other species, germination percentage is unaffected regardless of whether the seeds pass through the gut^37^,^38^. In the present study, the lower germination percentage of SFD and SID of *E. excelsum* compared with that of SFF and SIF at each storage period under both dry and wet storage conditions indicated that seeds that underwent gut passage exhibited decreased germinability during storage. Similar results on fresh seeds were found by Cosyns *et al.*^17^ who studied the effect of passage through the gastrointestinal tract on the seed germination of 19 plant species that occur in semi-natural grassland communities in Western Europe. Bravo *et al.*^39^ also found that seeds of *Solanum nigrum* ingested by great bustard (*Otis tarda*) had lower germination percentage than non-ingested seeds. Furthermore, other studies on seed dispersal effectiveness in geese also described a decrease in seed germination percentage after gut passage^40^,^41^. Thus, the results obtained in the present study do not support our hypothesis that seeds exhibit enhanced germination percentage during storage after gut passage. Changes in seed trait, including seed mass, water content, permeability, and texture, have been regarded to contribute to the heterogeneity in the germinability of fresh and stored seeds^25^. Furthermore, storage conditions influence seed vigor and germination patterns^33^,^42^. Given that the mechanism of the decrease in seed germination percentage after gut passage remains unknown^11^, we can reasonably speculate that the seed germination percentage of *E. excelsum* decreased in the present study as a result of the combined effect of storage conditions and seed trait modification after frugivore gut passage. An excellent work conducted by Traveset *et al.*^16^ found that seed passage through bird gut can increase seed permeability, which improves the germinability of fresh seeds. However, this change may also be detrimental because it may increase the possibility of embryo harm and pathogenic attack, which can cause seed mortality when seeds are under cold storage conditions^43^,^44^,^45^. Furthermore, SIF of *E. excelsum* under dry storage conditions had the highest germination percentage regardless of the storage period, suggesting that the existence of pulp can protect the seeds from harm during cold storage and improve their germinability. Although we did not examine the specific changes in seed traits of *E. excelsum,* the highest germination percentage of SIF supported our former speculation to a certain degree.

Passage through frugivore gut could change the seed germination rate^46^,^47^. Seed germination time is related to the fate of seedling establishment and survival^48^; early germination generally enhances competition and plant fitness^49^,^50^. In this study, the SGT and MGT of seeds after gut passage (SFD and SID) were lower than those of SFF and SIF, regardless of the other storage conditions. This finding indicates that gut passage accelerates the germination speed of *E. excelsum* during storage. Given that the increase in germination speed after frugivore gut passage is a recurrent result in most studies^27^, our results also support the findings of previous studies that fresh-fruit species have higher germination rates after gut passage than those that do not pass through the gut^26^,^48^. Seed coat modification is most often induced when comparing the germination rate between ingested and un-ingested seeds of the same species. Traveset *et al.*^16^ found that seeds of *Phillyrea angustifolia* that passed through bird guts have thinner coat and subsequently become more permeable to water, which directly accelerates germination. A decrease in seed-coat thickness and an increase in germination rate after passing through frugivore digestive tracts was also found in studies on *Rubia fruticosa*^51^. In addition, Paulsen and Högstedt argued that seeds that underwent gut passage show mechanical and chemical scarification of the seed coat, which may enable the hypocotyl to emerge easily from the seed, reducing the germination energy cost and contributing to accelerated germination rate^48^,^52^. Seed retention time in frugivore gut is also an important factor that influences germination rate. Traveset *et al.*^26^ reported that small seeds retained for a longer time in the digestive tract show enhanced germination rate. The seeds of *E. excelsum* are small, with a 1000-seed mass of 0.85 ± 0.05 g. The retention time during the passage of seeds through the gut of *P. xanthorrhoea* and *P. jocosus* ranges from 25 min to 40 min (unpublished data), which is partly consistent with other small-seeded plant species (such as *Sorbus ancuparia*) that showed improved germination rate after frugivore gut passage^48^. Thus, we believe that the increased seed germination rate of *E. excelsum* after gut passage during storage may have resulted from changes in seed traits while in the process of frugivore gut digestion. This assumption, however, needs further investigation.

The interaction of seed traits with storage conditions may influence their vigor during storage, and thereby affect seed germinability and plant performance^42^. In this study, the four types of *E. excelsum* seeds, especially seeds of SIF, germinated more successfully than fresh seeds^29^ at 1 and 3 months after storage. In addition, the speed of germination (SGT and MGT) significantly increased after 3 and 6 months of storage. Fresh seeds generally germinate under specific limited conditions, and storage can gradually remit such conditions that are beneficial for germination^53^,^54^. Seeds stored under cold dry conditions have a higher germination rate than fresh seeds of many species, such as *Salsola imbricata*, *Haloxylon salicornicum*, and *Onopordum acanthium*^54^,^55^. Thus, our results supported that short duration of cold dry storage reduces the requirements during seed germination.

Although the specific mechanism was not determined in the present study, we demonstrated that seeds of *E. excelsum* that passed through frugivore gut have significantly lower final germination percentage and faster germination time during storage. We also found that the presence of pulp can protect the seeds from harm during cold dry storage and significantly enhance germination, which is inconsistent with the findings of previous studies on fresh seeds^56^. These results suggest that frugivore gut passage have long-term impact on the viability and germination performance of the seeds. Although we still do not know how general these results would be for other plant species, we may expect that this finding can be extrapolated to other species because of the recognized effects of frugivore gut passage and storage on seed viability. However, given that numerous studies have found that the effect of frugivore gut passage on seed germination (both percentage and rate) varies among plant and disperser species, as well as the type of experimental conditions, the species-specific effects of dispersers on seed storage should be tested under a wide range of both plant and disperser species in future experiments. Furthermore, given the seed viability during storage reflecting the capacity of the species to maintain the soil seed bank^31^,^32^, we may conclude that seeds of *E. excelsum* that passed through frugivore gut cannot form persistent soil seed bank in the field. However, experimental field studies disentangling the importance of frugivore gut seed passage and storage on soil seed bank formation, seedling recruitment, and long term germplasm conservation deserves further attention.

## Methods

### Study species

*Euryodendron excelsum* is an endangered plant from the monotypic genus of *Euryodendron* belonging to the family *Theaceae*, which is endemic to southern China. It has been ranked as a second-category protected plant in the Red List of China and listed as a critically endangered plant based on the species categories of the International Union for Conservation of Nature and Natural Resources (IUCN) because of its limited distribution and small population^28^,^29^.

*E. excelsum* is an evergreen tree that grows up to a height of 25 m, with a trunk diameter of 1.5 m. Its flower is small, white, and bisexual, with approximately 5 mm in diameter. The species is propagated only by its seeds. *E. excelsum* produces abundant flowers during its flowering stage in late September to early November. Its fruiting period is between October and February. Mature seeds are small and nephroid-shaped; the seeds have no dormancy. The seed dispersion of *E. excelsum* is barochory and ornithochory in nature. According to our previous field survey, *Pycnonotus xanthorrhoea* and *P. jocosus* are the main birds that eat the fruits of *E. excelsum* in daytime. Birds can excrete seeds shortly after consumption, and the passage of seeds through their gut does not affect the final germination percentage of fresh fruit seeds^28^,^29^,^30^.

### Study site and seed collection

Thus far, only a single remnant population of *E. excelsum* with a highly isolated and fragmented distribution exists in the Bajia region, Yangchun County of Guangdong Province. Bajia has a humid subtropical monsoon climate, with a mean annual temperature of 22 °C and annual of more than 2000 mm. Given the high frequency of anthropogenic disturbance, *E. excelsum* mainly exists in two community types: secondary evergreen broad-leaved forests and artificial forests^28^.

Samplings were collected manually from the remnant population on 6 January 2009 from the Bajia region (21°57′N and 111°24′E). To obtain the ripened fruits of *E.excelsum* and bird droppings in the same time period, we first cleaned the ground under the adult individuals in the morning, and then collected dropped fruits and bird droppings in the evening. Fruits and bird droppings were stored in sealed polyethylene bags and transported to the laboratory (Yunnan University) within 24 h after collection.

### Seed storage and germination tests

Four types of seeds, including seeds separated from fruits (SFF), seeds separated from droppings (SFD), seeds in intact fruits (SIF), and seeds in bird droppings (SID), were prepared to determine seed storage behavior. SFF and SFD were extracted by hand from fresh fruits and droppings, respectively. Seeds of *E. excelsum* in bird droppings were identified on the basis of shape and color. Seeds were stored under dry and wet conditions. For wet storage, the four types of seeds were packed in gauze bags and buried in wet sand placed inside hermetic glass vials. For dry storage, the seeds were stored directly inside hermetic glass vials. The glass vials were stored for 1, 3 and 6 months at 4 °C.

After storage, the seeds were initially washed with tap water to manually remove appendages (sand, pulp, or faecal material), surface sterilized with 0.85% sodium hypochlorite for 1 min, and subsequently washed with distilled water. The seeds were then placed on a two-layer filter paper moistened with distilled water in glass Petri dishes (diameter, 9 cm). The number of germinated seeds was recorded daily, and the seeds were considered to be germinated when the length of the radicle was ≥2 mm. Distilled water was added as required to ensure that moisture was not limited during the germination tests. The experiment was terminated when no seeds had germinated for 7 days, and the germinated seeds were removed from the dishes to avoid any potential influence on the remaining seeds. Each germination test was conducted at a 12 h light period at 20 °C, using four replicates of 25 seeds each.

### Statistical analysis

The germination percentage was calculated using the following equations: germination percentage (%) = (N/S) × 100%, where N is the total number of germinated seeds and S is the total number of seeds. This variable was considered a surrogate of seed germinability in the test. Mean germination time (MGT) and start germination time (SGT) were calculated to assess the rate of germination^57^ using the following equations: MGT 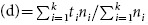, where *t*_*i*_ is the *i*th day from the start of the experiment, *n*_*i*_ is the number of germinated seeds at the *i*th day from the start of the experiment, and *k* is the time of the last germination. The time to start germination (SGT) was calculated as the day when the first seed germinated in each replicate. Smaller values of MGT and SGT corresponded to faster germination rates (i.e., an early start of germination).

The effect of storage conditions on the germination percentage and rate was determined using general and generalized linear models (GLMs). Binomial error distributions were fitted to analyze the germination percentage, while Gaussian error distributions were used for models with germination rate (SGT and MGT). In all models, storage periods, methods, and seed types were used as independent categorical variables, with all interaction terms included. The best model for each of the three dependent variables (germination percentage, SGT, and MGT) was determined by calculating the corrected Akaike’s Information Criteria for small sample size (AICc), log-likelihood (LL), relative model differences in AICc (ΔAICc), and relative model weights (*wi*) using the *aictab* function of the *AICcmodavg* library. Models with low ΔAICc and high Akaike weights (*w*_*i*_, interpreted as probabilities) were considered to have the greatest statistical support. An ΔAICc value lower than 5, indicates a model having substantial support, as indicated by Anderson^58^. All statistical analyses were conducted under the open source environment R.

## Additional Information

**How to cite this article**: Shikang, S. *et al.* Does the passage of seeds through frugivore gut affect their storage: A case study on the endangered plant *Euryodendron excelsum*. *Sci. Rep.* **5**, 11615; doi: 10.1038/srep11615 (2015).

## Figures and Tables

**Table 1:** Generalised linear models (GLMs) analysis of the different storage conditions and their interactions on the germination traits of *E. excelsum* seeds (*** P < 0.001; ** P < 0.01; * P < 0.05)

**Variables**	**Germination traits**						
	**Germination percentage**			**SGT**		**MGT**	
	**df**	**F**	**Pr(>F)**	**F**	**Pr(>F)**	**F**	**Pr(>F)**
Storage periods	1	28.5617	8.351e-07***	14.170	0.000317***	16.597	0.000108***
Methods	1	9.1826	0.003290**	1.286	0.260243	3.818	0.054200
Seed types	3	9.4543	2.037e-05***	1.821	0.149903	4.871	0.003663**
Storage periods *Methods	1	9.3813	0.002986**	0.246	0.621116	0.000	0.993405
Storage periods *Seed types	3	4.5695	0.005257**	0.003	0.999822	0.143	0.933935
Methods*Seed types	3	4.4132	0.006343**	0.077	0.972394	0.472	0.702325
Storage periods *Methods* Seed types	3	3.7054	0.014947*	0.096	0.961965	0.375	0.771448

**Table 2:** Models examining the importance of storage periods (1, 3 or 6 month), storage methods (dry or wet), and seed types (seeds separated from fruits, seeds separated from droppings, seeds in intact fruits, and seeds in bird droppings) on the germination percentage, mean germination time (MGT) and start germination time (SGT)

**Response variable**	**Model parameters**	***K***	***LL***	**AIC**_**c**_	**ΔAIC**_**c**_	***w***_***i***_
Germination percentage	Storage periods * Methods * Seed types	16	−216.34	471.56	0.00	1.00
MGT	Storage periods + Methods + Seed types	7	−263.57	542.42	0.00	0.71
	Storage periods + Seed types	6	−265.73	544.41	1.99	0.26
SGT	Storage periods	3	−261.71	529.68	0.00	0.31
	Storage periods + Seed types	6	−258.61	530.17	0.49	0.24
	Storage periods + Methods	4	−261.00	530.43	0.76	0.21
	Storage periods + Methods + Seed types	7	−257.85	530.98	1.31	0.16
	Storage periods* Methods	5	−260.86	532.39	2.71	0.08

Models are ordered by corrected Akaike information criterion (AICc) scores for small sample sizes, and only those with a ΔAICc ≤ 5 are shown. The symbol * indicates interaction and + indicates an addition. Model-selection parameters include the number of parameters (*K*), log-likelihood (*LL*), *AIC*_*c*_ scores, relative model differences in AIC_c_ (ΔAIC_c_), and relative model weights (*w*_*i*_).
